# Sudden Onset Acral Pigmented Macules: An Innocuous Diagnosis

**DOI:** 10.5826/dpc.1103a54

**Published:** 2021-07-08

**Authors:** Rashmi Jindal, Payal Chauhan, Robin Chugh

**Affiliations:** Department of Dermatology, Venereology & Leprosy, Himalayan Institute of Medical Sciences, Swami Rama Himalayan University, Dehradun, Uttarakhand

**Keywords:** Cydnidiae, burrowing, acral, pigmented, macules

## Case Presentation

**Figure 1:**
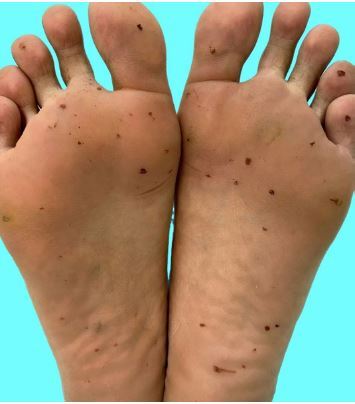
Multiple non-blanchable and non-tender brown-black macules over soles.

**Figure 2:**
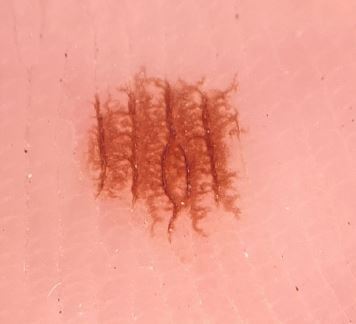
Streaks of orange-brown pigment with ridge enhancement on dermoscopy (DermLite DL2 Hybrid, × 10).

A 60-year-old healthy man presented with sudden onset of asymptomatic, brown-black macules over his feet soles. Lesions were randomly distributed with relative sparing of instep, pinpoint to a few millimeters in size, non-blanchable, and non-tender (Figure 1). Attempts to wipe them off with alcohol swab were unsuccessful. Dermoscopy (DermLite DL200 hybrid, ×10, 3Gen, San Juan Capistrano, California) revealed streaks of orange-brown pigment with ridge enhancement (Figure 2). The patient had the habit of walking barefoot in his dairy shop, surrounded by ample foliage, and had noticed low flying winged insects during this rainy season. A final diagnosis of Cydnidae (burrower bug) pigmentation was established, supported by a history of sudden onset of asymptomatic lesions and dermoscopic examination.

## Teaching Point

**Figure 3:**
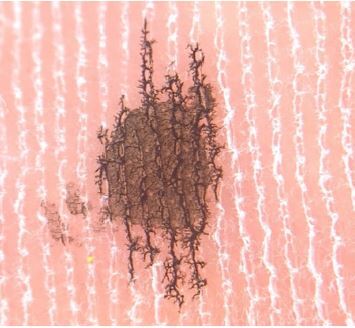
Replication of ridge enhancement with black fountain pen ink on dermoscopy.

Pigmented macules have been previously reported in India due to burrower bug *Chilocoris* spp (family, *Cydnidae*; super-family, P*entatomoidea*) [1,2]. These otherwise innocuous bugs live inside the soil, feed on the roots of underground plants, and release an orange-brown pigmented substance as a defense mechanism, that stains the skin when accidentally crushed. The pattern of streaks with ridge enhancement is due to seeping of this pigmented substance into the ridges of feet soles as replicated by application of a drop of black fountain pen ink (Figure 3). To the unwary, these pigmented macules may cause concern and could appear associated with syndromic lentiginosis or a viral hemorrhagic fever manifestation, resulting in unnecessary investigations.
